# Editorial: Light-mediated regulation of plant physiology

**DOI:** 10.3389/fpls.2024.1531410

**Published:** 2025-01-03

**Authors:** Andres Romanowski, Elena Monte, Carlos Esteban Hernando, Gabriela Toledo-Ortiz, John M. Christie, Jordi Moreno-Romero, Karen J. Halliday

**Affiliations:** ^1^ Laboratory of Molecular Biology, Wageningen University and Research, Wageningen, Netherlands; ^2^ Centre for Research in Agricultural Genomics (CRAG), CSIC-IRTA-UAB-UB, Cerdanyola del Vallès, Barcelona, Spain; ^3^ Instituto de Investigaciones Bioquimicas de Buenos Aires (IIBBA) - Consejo Nacional de Investigaciones Científicas y Técnicas (CONICET), Ciudad Autonoma de Buenos Aires, Argentina; ^4^ School of Molecular Biosciences, College of Medical, Veterinary and Life Sciences, Bower Building, University of Glasgow, Glasgow, United Kingdom; ^5^ Departament de Bioquimica i Biologia Molecular, Universitat Autonoma de Barcelona (UAB), Bellaterra, Barcelona, Spain; ^6^ Institute of Molecular Plant Sciences, School of Biological Sciences, University of Edinburgh, Edinburgh, United Kingdom

**Keywords:** photomorphogenesis, phytochrome, miRNA, pigment biosynthesis, splicing, translation, circadian clock, light signalling

The field of plant photobiology has evolved rapidly over the past decade, expanding our understanding of how light influences every aspect of plant life, from gene expression to ecosystem interactions. This Research Topic aims to update the current knowledge on the intricate mechanisms underlying light-driven growth and metabolic/environmental adaptation processes, offering insights into the cellular complexity that underpins plant adaptability to varying light conditions.

Here, we highlight the primary contributions of these studies, grouped into four major themes that encompass a broad perspective on light-mediated regulation in plants.

## Regulatory layers in photomorphogenesis: phytochromes, microRNAs, and pigment biosynthesis

Photomorphogenesis, the developmental transition from dark to light conditions, involves multiple regulatory layers, including (but not limited to) light perception, signalling and changes in gene expression and translation. Cota-Ruiz et al. explored the interplay between phytochromes and zinc finger proteins (ZFPs) in Arabidopsis, revealing that phytochrome-dependent regulation of hypocotyl elongation can be modulated through ZFPs. This study provides insights into how phytochromes orchestrate complex molecular mechanisms that control light-mediated development. Lakatos et al. delved into the role of RNA-induced silencing complex (RISC)-loaded microRNAs in Arabidopsis de-etiolation, showing that while RISC loading impacts active miRNA formation in organ-specific pools, it has a limited role in global miRNA regulation during photomorphogenesis, as total levels and the RISC-loaded miRNAome show minimal changes. However, there is one notable exception: while miR163 is present at low levels in the dark, it sharply increases in abundance and RISC loading efficiency when seedlings are shifted to light. Furthermore, increased miR163 expression is required for normal photomorphogenesis. This study demonstrated the existence of specialized miRNA regulatory mechanisms for fine-tuning specific responses. Xie et al. employed transcriptomic and metabolomic analyses to identify key genes and transcription factors involved in light-regulated anthocyanin biosynthesis in *Perilla frutescens.* These findings have implications for medicinal and ornamental applications. Together, these studies emphasise the importance of multiple molecular layers in mediating photomorphogenesis.

## The influence of light quality and quantity on plant development

Light quality, encompassing different wavelengths, plays diverse roles in the regulation of distinct developmental and physiological processes in plants. Tang et al. demonstrated how specific wavelengths, such as red and blue light, impact both the transcriptome and metabolome of *Mesona chinensis*, with red light markedly boosting growth-related traits. This study highlights the importance of combining transcriptomic and metabolomic data to determine the regulatory effects of light on plant development. Zioutopoulou et al. revealed that Ultraviolet radiation B (UV-B) radiation could trigger photoperiodic flowering in Arabidopsis via the UV-B resistance 8 (UVR8) pathway, underscoring its role in modulating the vegetative to reproductive transition. Sheridan et al. further examined UV-B exposure, showing its specific influence on primary root elongation by directly affecting meristematic cell proliferation in Arabidopsis, an indication of light’s spatial specificity in plant tissues. Berardi et al. demonstrated the existence of a novel signalling pathway involving increased production of singlet oxygen in response to low red-to-far-red ratios, contributing to a greater understanding of the complexity behind shade avoidance strategies in Arabidopsis. Beyond quality, the intensity or quantity of light also plays a role. Serrano-Pérez et al. investigated the molecular mechanisms underlying high light resistance in the charophyte alga *Klebsormidium nitens*, uncovering ancient photoprotective mechanisms that may have been crucial for plant terrestrialisation. Together, these studies have revealed the complex and multifaceted roles of both light quality and quantity in shaping plant growth and metabolism.

## Light-mediated mechanisms of regulation of gene expression: splicing and mRNA translation

In plants, light exerts control over gene expression at the post-transcriptional level through splicing and mRNA translation. Ma et al. explored the impact of light on alternative splicing in *Artemisia annua*, showing that light-induced intron retention could regulate sesquiterpenoid biosynthesis. Schwenk et al. identified a novel cytoplasmic function of phytochrome A in mediating far-red light-induced disassembly of processing bodies in Arabidopsis, leading to the release of translationally halted mRNA. Together, these studies enrich the understudied field of post-transcriptional and translational regulation of gene expression, shedding light on the nuanced control mechanisms employed by plants to modulate gene activity.

## Interplay of circadian rhythm with light and stress in plants

Circadian rhythms enable plants to anticipate daily environmental changes. Light is a key modulator of this internal clock. Rhodes et al. uncovered the dual role of Far-Red Elongated Hypocotyls 3 (FHY3) in light input to the circadian clock, revealing a red-light-specific disruption of rhythmicity in *fhy3* mutants and suggesting an integrative role for FHY3 in the red/blue light ratio in clock regulation. Jurca et al. demonstrated that the blue light receptor ZEITLUPE (ZTL) modulates Abscisic acid (ABA)-induced stomatal closure, a critical adaptation under drought stress. These studies illustrate the intricate linkages between light signalling, circadian rhythm, and stress responses, highlighting the adaptive advantages conferred by these interconnected systems.

## Closing remarks

Light has a pervasive function in plant biological processes. This is evidenced in the research presented in this Research Topic, which reveals light’s intricate and versatile roles in plant development, physiology, and stress response across various species, including the model plant *Arabidopsis*. By employing different techniques, including transcriptomic, metabolomic, and physiological approaches, these studies provide valuable insights into the molecular frameworks underlying light-mediated plant responses, revealing the interconnected nature of light signalling pathways across different biological levels. This Research Topic advances our fundamental understanding and opens new avenues for applied research, with implications for agriculture and plant-based industries.

